# Evidence for prehistoric origins of the G2019S mutation in the North African Berber population

**DOI:** 10.1371/journal.pone.0181335

**Published:** 2017-07-19

**Authors:** Rafiqua Ben El Haj, Ayyoub Salmi, Wafa Regragui, Ahmed Moussa, Naima Bouslam, Houyam Tibar, Ali Benomar, Mohamed Yahyaoui, Ahmed Bouhouche

**Affiliations:** 1 Research Team in Neurology and Neurogenetics, Genomics Center of Human Pathologies, Medical School and Pharmacy, Mohammed V University, Rabat, Morocco; 2 Laboratory of Information and Communication Technologies, National School of Applied Sciences, Abdelmalek Essaadi University, Tanger, Morocco; 3 Department of Neurology and Neurogenetics, Specialties Hospital, IBN Sina University Hospital Center, Rabat, Morocco; University College London Institute of Neurology, UNITED KINGDOM

## Abstract

The most common cause of the monogenic form of Parkinson’s disease known so far is the G2019S mutation of the *leucine-rich repeat kinase 2* (*LRRK2*) gene. Its frequency varies greatly among ethnic groups and geographic regions ranging from less than 0.1% in Asia to 40% in North Africa. This mutation has three distinct haplotypes; haplotype 1 being the oldest and most common. Recent studies have dated haplotype 1 of the G2019S mutation to about 4000 years ago, but it remains controversial whether the mutation has a Near-Eastern or Moroccan-Berber ancestral origin. To decipher this evolutionary history, we genotyped 10 microsatellite markers spanning a region of 11.27 Mb in a total of 57 unrelated Moroccan PD patients carrying the G2019S mutation for which the Berber or Arab origin was established over 3 generations based on spoken language. We estimated the age of the most recent common ancestor for the 36 Arab-speaking and the 15 Berber-speaking G2019S carriers using the likelihood-based method with a mutation rate of 10^−4^. Data analysis suggests that the shortest haplotype originated in a patient of Berber ethnicity. The common founder was estimated to have lived 159 generations ago (95% CI 116–224) for Arab patients, and 200 generations ago (95% CI 123–348) for Berber patients. Then, 29 native North African males carrying the mutation were assessed for specific uniparental markers by sequencing the Y-chromosome (E-M81, E-M78, and M-267) and mitochondrial DNA (mtDNA) hypervariable regions (HV1 and HV2) to examine paternal and maternal contributions, respectively. Results showed that the autochthonous genetic component reached 76% for mtDNA (Eurasian and north African haplogroups) and 59% for the Y-chromosome (E-M81 and E-M78), suggesting that the G2019S mutation may have arisen in an autochthonous DNA pool. Therefore, we conclude that *LRRK2* G2019S mutation most likely originated in a Berber founder who lived at least 5000 years ago (95% CI 3075–8700).

## Introduction

Parkinson’s disease (PD) is the most common neurodegenerative disorder after Alzheimer’s disease, affecting about 1–2% of people over 60 years of age [1]. PD is characterized by clinical features that comprise a large spectrum of motor symptoms including, resting tremor, rigidity and bradykinesia. These manifestations are associated with a severe and progressive degeneration of dopaminergic neurons within the substantia nigra *pars compacta* [2].

In the past two decades, 15 genes have been described for inherited forms of PD. Among them, there are at least 5 confirmed genes responsible for autosomal dominant forms of PD: *SNCA* (PARK1/4), *LRRK2* (PARK8), *VPS35* (PARK17), *DNAJC13* and *CHCHD2* [3,4]. Mutations in the *LRRK2* gene are the most frequently identified as monogenic causes of PD. Indeed, they have been found in about 1.8% of healthy controls, 3.6% of sporadic PD cases and 10% of autosomal dominant familial PD cases [1]. Approximately 80 variants have been identified in *LRRK2* gene so far [5]. Of these variants, 7 mutations are thought to be pathogenic but the most common one remains the G2019S mutation, which accounts for 5–6% of familial and 1–2% of sporadic PD cases [1,6]. Intriguingly, the frequency of this mutation varies widely depending on geographic origin and ethnicity. Nevertheless, the highest frequencies are those recorded in Ashkenazi Jews and North African populations reaching up to approximately20% and 40% of PD patients respectively [7].

Several studies on different populations around the world attempted to define the origin of the *LRRK2* G2019S mutation and date the age of emergence. Presently, the mutation is associated with three different haplotypes. While haplotype 2 has been found in individuals of European descent, haplotype 3 was first identified in Turkish and Japanese families. These two haplotypes have not been well studied and seem to be of more recent origin than haplotype 1[8–11]. Despite being widespread across the world, haplotype 1 follows from a common ancestral founder [12]. A study on a European cohort estimated the common founder occurrence to 725 years ago [13], though a subsequent study on Ashkenazi, European and European-American families has estimated it to 2250 years ago; a period that overlaps with the Jewish Diaspora from 586 BC to 70 AD [9]. In addition, Warren et al. [14] dated the most recent common ancestor of patients with haplotype 1 from Tunisia, America, European, and Middle-Eastern countries to 2600 years ago. Furthermore, Bar-Shira et al. [15] estimated that the common ancestor of Ashkenazi Jewish G2019S carriers lived about 1830 years ago. However, the most significant study carried out by Lesage et al. on a large sample of patients belonging to several ethnicities, which suggested that haplotype 1 originated in a Near-Eastern founder at least 4000 years ago [11]. Finally, a recent study on North African patients concluded that the G2019S mutation originated in a Moroccan Berber patient who lived 3840 years ago [16].

While the *LRRK2* G2019S mutation is largely widespread throughout the North African populations, which are composed of two predominant ethnic groups Berbers and Arabs. It should be noted that in all these previous studies, the ethnic origin of this population was considered as Arab or Arab-Berber without a clear distinction between the two ethnic groups, or not documented at all. In addition to having a small sample of Moroccan patients, this discrepancy places further limitations on both the power and clarity of experimental analyses in previous studies. To better distinguish between these ethnic backgrounds, we employ three generations of spoken language as a tool of differentiation. In the present study, we aim to date the age of the most recent common ancestor of the G2019S carriers within Moroccan PD patients and to determine whether the mutation is of Berber or Arab origin by studying maternal and paternal lineages using both mitochondrial and Y-chromosomal genetic markers.

## Subjects and methods

### Patients and controls

A total of 57 unrelated PD patients with the G2019S mutation were selected from the Department of Neurology and Neurogenetics (Specialties Hospital, Rabat, Morocco). All patients agreed to participate and were recruited from October 2013 to June 2016 according to criteria described earlier [17]. Age at examination ranged from 35 to 86 years old and the mean age was 59 ± 11.4 years. In addition, 77 control individuals with no family history of neurological disease were recruited at the Blood Transfusion Center (Rabat, Morocco). All individuals, patients and controls included in the study, are native Moroccans and their ethnical origin was determined on the basis of spoken language (Berber or Arab) through three generations. Participants were classified as Arab- or Berber-speaking if all of the individuals’ grandparents speak Arab or Berber languages respectively, and mixed if at least one grandparent speaks a different language. This study was approved by the biomedical research ethics committee of the Medical School and Pharmacy of Rabat (CERB), and written informed consent was obtained from all subjects.

### Genotyping and estimation of the age of the G2019S mutation

Genomic DNA was extracted from peripheral blood leukocytes using Isolate II Genomic DNA kit from Bioline. Haplotypes were constructed using the following microsatellite markers: D12S1648, D12S2080, D12S2194, D12S2514, D12S2516, D12S2518, D12S2519, D12S2520, D12S1048 and D12S1301, covering an interval of 11.27Mb. The genotyping of microsatellite markers was performed using fluorescently labeled primers, and the products were pooled and analyzed on a 310 Genetic Analyzer using Genotyper Software version 3.7 (Applied Biosystems). The CEPH DNA sample 1331–01 was used as external standard to control for consistency between runs. We used Phase v2.1.1 software [18] to infer the haplotypic phase for all subjects. The age of the most recent common ancestor of the 36 Arab-speaking and the 15 Berber-speaking G2019S carriers was estimated separately using a likelihood-based method implemented in the ESTIAGE program [19]. We used allele frequencies obtained from the control individuals and a stepwise mutation model with a mutation rate of 10^−4^ at each marker per generation was assumed [20].

### Analysis of uniparental markers

Since the G2019S mutation can be transmitted by either parent, and the uniparental markers can only be studied simultaneously in males, the 29 males carrying the G2019S mutation (18 Arab-speakers, 8 Berber-speakers and 3 mixed) were studied for their Y-chromosome and mtDNA markers. These markers are known to be inherited unchanged from one male or female generation to the next unless mutations occur.

The biallelic markers E-M81 and E-M78, reported as being the specific male lineage of autochthonous Berbers of North Africa [21,22] and J1-M267, the most prominent genetic marker of males from the Levantine [23,24], were analyzed in the 29 male carriers. All markers were amplified by PCR using primers as described previously [25,26]. For the maternal lineages, we amplified the two mtDNA hypervariable regions, HV1 and HV2, using primers F15971/R16451 for HV1 and F15/R484 for HV2 as described by Levin BC et al. (1999) [27]. All PCR fragments were sequenced using Big Dye Terminator Cycle Ready Reaction 3.1 Kits and sequence analysis was done through an ABI 3130xl sequencer. Data were analyzed by SeqScape2.1 software (Applied Biosystems, Foster City, CA). The mtDNA haplogroup assignment was done using the HaploGrep2 software based on the underlying classification tree PhyloTree Build 17 [28].

## Results

### Genotyping and estimation of the age of the G2019S mutation

Among the 57 unrelated PD patients carrying the *LRRK2* G2019S mutation, 29 were males and 28 were females. Of these patients, 52 were heterozygous and 5 were homozygous for the mutation. Their ethnic origin was investigated over three generations on the basis of their spoken language (Berber or Arab). Surprisingly, we found that 63% (36/57) of patients with the G2019S mutation were from Arab-speaking families and only 26% (15/57) were from Berber-speaking families for at least three generations. In the six remaining families (11%), the patients’ grandparents were found to speak both languages, and were therefore considered to be of mixed descent.

The 36 Arab-speaking and 15 Berber-speaking patients were genotyped for 10 microsatellites around the G2019S mutation; patients of mixed origin were excluded because it is not possible to determine precisely the parent from whom the mutation was inherited, since their DNA was not available for analysis. The shared haplotype was inferred from patients homozygous for the G2019S mutation (Table 1). This haplotype, covering a distance of 575 Kb and spanning microsatellite markers D12S2194 to D12S1048, corresponds with the most frequent haplotype obtained by PHASE software. This finding only differs from previous studies in that the inferred haplotype concerns the shared allele at marker D12S2194, which was 261bp in our study instead of 257bp. In addition to the fact that 80% (4/5) of patients homozygous for the G2019S mutation were also homozygous for the 261bp allele, this particular allele was also found in 48% (49/102) of chromosomes in PD patients and in only 8% (13/154) of chromosomes in control individuals; whereas the frequency of the 257bp allele was 18% (18/102) and 20% (31/154) for these groups, respectively (S1 and S2 Tables). The use of the 261bp allele at D12S2194 reduced the number of recombination events at this locus and thus enabled a more accurate estimation of its age.

**Table 1:** Shared haplotype inferred from five G2019S homozygous patients.

Marker	Patients ID					Shared haplotype
	3332	3561	3601	3665	3811	
D12S1648	112/108	120/112	108/124	104/128	108/112	
D12S2080	188	180/188	180	180	180/188	
D12S2194	261	261	261	261	261/257	**261**
D12S2514	291	291	291	291	291	**291**
D12S2516	254	254	254	254	254	**254**
G2019S	A/A	A/A	A/A	A/A	A/A	**A/A**
D12S2518	154	154	154	154	154	**154**
D12S2519	132	132	132	132	132	**132**
D12S2520	260	260	260	260	260	**260**
D12S1048	214	214/223	214	214	214	**214**
D12S1301	110	110	110/114	102	94/98	

Interestingly, 75% (38/51) of G2019S carriers showed the shared haplotype of 575 kb in length, which corresponds with the already reported MENA haplotype and is referred to as haplotype 1, indicating that they shared a common ancestor. The shortest haplotype was found in a patient of Berber origin (S1 Table). We estimated the age of the common ancestor of the 51 Moroccan G2019S carriers to 174 generation ago (95% CI 133–230). If a generation is defined as 25 years, then this corresponds to 4350 years (95% CI 3325–5750). The common founder was estimated to have lived 159 generations ago (95% CI 116–224) for the 36 G2019S Arab-speaking carriers and 200 generations ago (95% CI 123–348) for the 15 G2019S Berber-speaking patients, corresponding to 3975 (95% CI 2900–5600) and 5000 years (95% CI 3075–8700) respectively (Table 2).

**Table 2:** Age estimation of the G2019S mutation using an intergenerational time interval of 25 years.

Patients	Number of generations	Age estimation
Arab-speaking (N = 36)	159 (95% CI 116–224)	3975 (95% CI 2900–5600)
Berber-speaking (N = 15)	200 (95% CI 123–348)	5000 (95% CI 3075–8700)
All (N = 51)	174 (95% CI 133–230)	4350 (95% CI 3325–5750)

### Analysis of uniparental markers

In Table 3, results of the Y-chromosome marker analysis show that the E-M81 was the most frequent haplogroup with a prevalence of 48% (14/29), whereas E-M78 and J1-M267 were found in 10% (3/29) and 14% (4/29) of male carriers, respectively. The paternal genetic components of native Berbers (E-M81 and E-M78) represented 87.5% (7/8) of Berber-speakers, 50% (9/18) of Arab-speakers, and 33% (1/3) of mixed patients, whereas the most prominent male lineage of the LevantineJ1-M267, represented 12.5% (1/8), 16,6% (3/18) and 0% (0/3) respectively.

**Table 3:** mtDNA and Y-chomosomeuniparental markers analysis for the 29 G2019S male carriers.

		Y-chromosome markers			
Patient	Langage	E-M81	E-M78	J1-M267	mtDNAhaplogroups
3076	A	-	-	-	U2e2
3336	A	+	-	-	I5a
3443	A	-	-	-	L2b
3450	A	-	-	-	N1a3a
3453	A	-	+	-	HV0f
3462	A	-	-	-	U6
3463	A	-	-	+	HV0
3540	A	+	-	-	L2c1a
3564	A	+	-	-	L3d1b1b
3589	A	-	-	-	L2a1b
3590	A	-	-	+	K2b1b
3639	A	+	-	-	U2
3665	A	+	-	-	HV0
3777	A	-	-	-	W5a
3783	A	+	-	-	U4a2a
3785	A	+	-	-	L3d
3787	A	-	-	+	H2a2a
3796	A	+	-	-	M1a5
3215	B	+	-	-	L2a1a
3280	B	-	+	-	H6
3331	B	+	-	-	B6
3393	B	-	-	+	H1e1a4
3434	B	+	-	-	U6
3445	B	+	-	-	K
3664	B	-	+	-	H6
3762	B	+	-	-	J1c3
3373	M	+	-	-	J2a2
3433	M	-	-	-	H13a2b3
3788	M	-	-	-	L2b1a

**A**: Arab-speaking;

**B**: Berber-speaking;

**M**: Mixed.

The maternal lineage was evaluated by sequencing the two mtDNA hypervariable regions, HV1 and HV2. Haplotypes and their haplogroup assignment for the 29 G2019S male carriers are presented in S3 Table and Table 3. The results show that haplogroups H, HV0, U (without U6), J, K, N1, and N2, belonging to the Eurasian lineages, were the most frequent haplogroups, overlapping with 66% (19/29) of male carriers. Among them, haplogroups belonging to the HV branch were the most common, comprising 28% (8/29). Haplogroups U6 and M1 of the North African lineages represented 10% (3/29), whereas sub-Saharan lineages (L2 and L3) were found in 24% (7/29) of our sample.

Furthermore, analysis of uniparental markers revealed that most males (13/29) carrying the G2019S mutation have both parents of Berber origin. The 4 male patients with the Arab J1-M267 marker have both of the native maternal lineage (mtDNA) haplogroups, H and K.

## Discussion

In this study, to determine whether the G2019S mutation has a Berber or an Arab origin in Morocco, the ethnic background of 57 unrelated Moroccan patients carrying the mutation was investigated. Surprisingly, the results suggest that the majority of patients with the G2019S mutation were from Arab-speaking families 63% (36/57) and only 26% (15/57) were from Berber-speaking families for the last three generations. Based on these relative frequencies, the G2019S mutation seems to be of Arab origin. If this is the case, however, one would expect to find the mutation at high frequencies in other Middle-Eastern Arab countries from where the Islamic conquest descended to Morocco and North Africa. The only studies on the G2019S mutation in Arab countries have come recently from Egypt, which reported a frequency among sporadic PD patients of 9.7% (11/113) in the north [29] and only 1.45% (1/69) in the South of the country [30], and also from Saudi Arabia and all Gulf countries where the mutation was reported to be absent [31,32]. Together, these data suggest that it is highly unlikely that the mutation is of Arabic origin.

To address this issue, we estimated the age of the most recent common ancestor in the 36 G2019S Arab-speaking patients and the 15 G2019S Berber-speaking patients separately by genotyping 10 flanking microsatellites. Using best-call haplotypes, we estimated the age of the common ancestor of the 36 Arab-speaking carriers with haplotype 1 to 159 generations ago (95% CI 97–161), and considering an intergenerational time interval of 25 years, this corresponds to about 3975 years. The common founder in the 15 G2019S Berber-speaking patients was estimated to have lived 200 generations ago (95% CI 123–348) corresponding to 5000 years (95% CI 3075–8700). These results, along with the fact that the shortest haplotype was observed in a Berber-speaking patient, indicate that the G2019S mutation might have arisen in Morocco for the first time in a Berber ancestor about 5000 years ago.

Our age estimation of the G2019S mutation in Berbers is older than what is reported in recent studies using the same study design. Also considering an intergenerational time interval of 25 years and using the ESTIAGE program, Lesage and collaborators reported an age estimate of about 3000 years using data collected from 67 North African patients and 8 microsatellites (D12S2080 to D12S1301) [11]. However, the age estimate provided by Lucotte et al. [16] was about 3200 years using 7 microsatellites (D12S2194 to D12S1048). In the first study, only 10 patients were of Moroccan origin and were considered Arabs, while in the second study, the authors focused on only 5 Berber- and 2 Arab-speaking patients of Moroccan origin, neither of which is representative of the Moroccan population. Our study, however, focuses on a large sample of 51 Moroccan patients for whom the Arabic or Berber ethnicities were evaluated on the basis of three generations of spoken language.

The estimated age of the G2019S mutation for Arab-speaking patients (3975 years) is older than the date of the Islamic conquest, which occurred 1300 years ago. This suggests that current Arab-speaking populations in Morocco are the result of interbreeding between Berbers and Arabs over many generations, and that the majority of people who declare themselves as Arabs are in fact Arabized Berbers [12,21,33]. Indeed, the Arab influence in the Maghreb was more cultural than a demographic replacement of the autochthonous Berber population. This hypothesis follows from the fact that the Arab invasion of the Maghreb in the 7th century was made by only few thousand people compared to millions of indigenous Berbers of the time [34], strengthening the hypothesis that the G2019S mutation occurred in a Berber founder. As an observation that lends additional support to this hypothesis is that the frequency distribution of the G2019S mutation along the coastal regions of the Mediterranean Basin and in Europe is correlated strongly with that of the Y-chromosome E-M81 haplogroup, which would have appeared in North Africa during the Neolithic period about 14200 years ago (www.yfull.com/tree/E-M81). This haplogroup, referred to as a genetic Berber marker, reaches a mean frequency of 42% in North Africa, with a decreasing gradient ranging from 98.1% in the southern and Berber regions of Morocco to 11.7% in the north of Egypt [21,22,35]. In the Middle East, this Berber marker was reported in 1.3% of Lebanese [36], 5% among Sephardic Jews, and 3.7% of Turkish [37], but it was absent in Arab countries [23,38]. In Europe, E-M81 haplogroup, widespread but rare, was found relatively higher in southern countries particularly in Spain and Portugal with the highest frequency found in Cantabria at 18.6% [39]. In agreement with our hypothesis, the community of Cantabria has also been reported to have the highest frequency of G2019S carriers at 8.7% [40].

In our sample of G2019S male carriers, the specific autochthonous Y-chromosome haplogroups, E-M81 and E-M78, which coalescence time has been estimated in North Africa to 15000 years [41], are the most prevalent and represent 58% of patients. These markers are found in 87.5% of Berber-speaking G2009S male patients and in 50% of Arab-speaking males, supporting the hypothesis of the Arabic cultural influence on Berbers, since the Arab conquest occurred six centuries ago. Indeed, the most prominent Arab Y-chromosome marker in Arabs, J1-M267 [23,24], has been found only in 14% of our G2019S male carriers. On the other hand, analysis of the maternal phylogeny of the males carrying the G2019S mutation revealed that their mitochondrial pool was characterized by a high frequency of West-Eurasian haplogroups (66%), a lower frequency of sub-Saharan L lineages (24%), and the presence of autochthonous haplogroups U6 and M1 (10%). This haplotype diversity of the G2019S carriers has been reported in similar proportions in the modern day Moroccan population [42–44] and also in Iberomaurusian skeletons unearthed from the archaeological site of Taforalt in Northeastern Morocco dating back to 12000 years [45], suggesting that the Moroccan mitochondrial pool has been more typical of the Mediterranean for at least the last 12000 years. When both uniparental markers are taken into account, our results showed that the autochthonous genetic components in our sample reached 76% for mtDNA (Eurasian and north African haplogroups) and 58% for the Y-chromosome (E-M81 and E-M78) which suggests that the G2019S mutation may have arisen in an autochthonous DNA pool.

**Fig 1:**
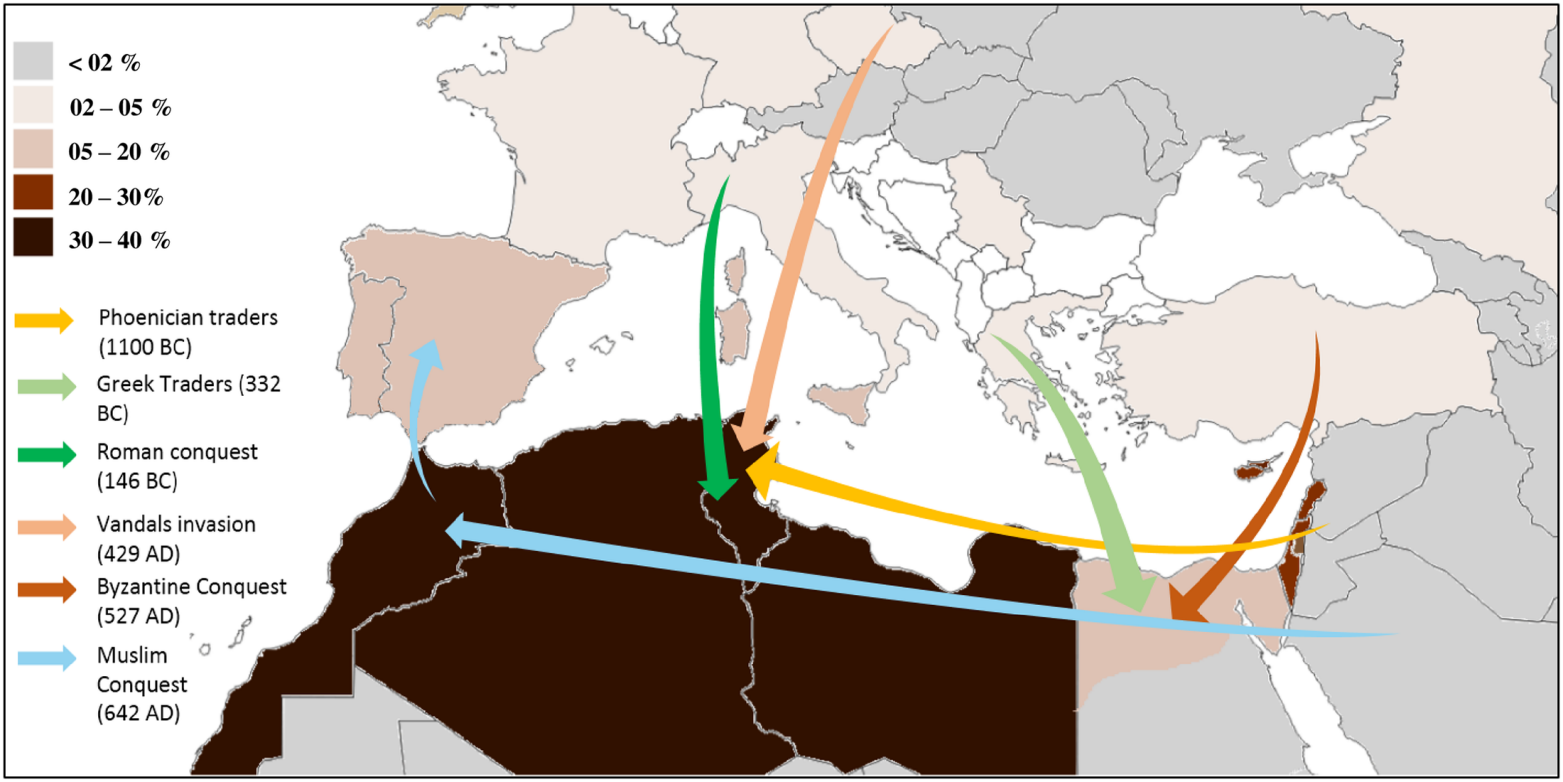
Map of the Mediterranean Basin illustrating the distribution of the G2019S mutation frequencies and the main conquests and invasions that the Maghreb has experienced since antiquity.

Since antiquity, the Berber people of the Maghreb have been invaded by many civilizations, all of which have been assimilated to various degrees: Phoenicians, Romans, Vandals, Byzantines and Arabs (Fig 1). Moreover, many Moors (Islamic Spaniards) and Jews arrived from Andalusia at the end of the 15th century. Despite this flux of humans into Maghreb area, the indigenous people have had genetic continuity since prehistoric time or even since the beginning of humanity, as the discovery in Morocco of the oldest *Homosapien* dating back to 300000 years supports [46].

The G2019S mutation is mainly found in Mediterranean populations with the highest frequencies seen in the Maghreb countries (Fig 1). Among them, Morocco presents with the highest prevalence and incidence of this mutation [17]. The general rule in population genetics stated that the geographic center of the origin of a mutation corresponds to the region in which the mutation is most frequent [47,48]. This conceptual framework, along with the results of uniparental markers, brings us to the most likely scenario for the origin of the G2019S mutation. This mutation would have appeared for the first time in a Berber founder living in the Maghreb at least 5000 years ago, with its center of dispersal located apparently further west, probably in Morocco. Over the course of several hundred years the mutation has increased its geographical range across the Northern coast of Africa as far as Egypt. The geographic distribution of G2019S frequencies in Europe (Fig 1), showing a north-south gradient with high values particularly in Spain and Portugal [11,12,40,49], could be explained by gene flow from Berber-Arab populations (mainly Berbers) into the Iberian Peninsula during the Muslim expansions from the 8th to 15th century[50]. Alternatively, it could also be caused by an earlier gene flow from Jewish populations, since their arrival was probably due to their migration from North Africa where they were already well-established. By genome-wide analysis, Campbell et al [51] showed that Jewish and non-Jewish North Africans formed distinct clusters, and that their mixture was not a recent event. Indeed, this event may go back to the Israelite’s arrival to the Maghreb by the earliest Phoenician traders. Despite this ancient connection, it is unlikely that the G2019S mutation is of Sephardic Jewish origin because the studies comparing Sephardic to Berber carriers all point to a Berber origin of the mutation. Berbers showed the highest prevalence and incidence of the mutation as well as the shorter haplotype compared to Sephardic Jews [16,50,52]. However, a problem arises when we attempt to explain the high frequency of this mutation in the Ashkenazim population. The G2019S mutation in Ashkenazim was reported to arise 4550 years (3250–6425) years ago [11] using a multi-ethnic ancestral haplotype. This age estimation, being slightly younger than that of our Berber ethnic group, is prior to the beginning of Jewish Diaspora and its establishment as an ethnic Jewish group. The presence of Jews in North Africa has been attested to as early as the Iron Age by the presence influence of memorial monuments allocated on architecture, practices and rituals to Jewish populations [53,54]. These Judeo-Berbers were reinforced by several waves of emigration, especially after the destruction of Jerusalem by Titus in 70 A.D.[55,56]. Judaism is thought to have spread among Berbers through proselytism, and its influence covered a significant portion of North Africa to the extent that it was even established as a state religion by the Berber Queen Kahina [57,58]. During the 7th century, some of these Judeo-Berbers would not have been converted to Islam by the Arab conquest and would have instead migrated to the Near-East as Jews, where some migrants might have served as Ashkenazi cofounders. Therefore, the high frequency of the G2019S mutation in the Ashkenazi population could be a result of isolation and genetic drift. Indeed, the origin of Ashkenazi Jews remains controversial, and their founders are thought to have lived in the Rhine Valley during the first millennium of the Common Era [59]. About 500 years ago, the size of the Ashkenazi population was estimated to consist of only several thousand people [60], but this period was followed by rapid population growth and a high rate of endogamy. Despite this consanguinity, it was reported that, due to Diasporas and migrations, the genome of the current Jewish people (including Ashkenazi) is very heterogeneous and has no specific genetic signature due to the influence of neighboring peoples [61]. This scenario gains support from the study at the genomic level by Henn BM and collaborators [62] who postulated that indigenous North African ancestry, spanning from the northwest to northeast, was most closely related to populations outside of Africa, and that their divergence from Near-Eastern and European groups preceded the Holocene for more than 12000 years. Therefore, it is important that further phylogenetic studies on G2019S carrier patients from other populations, Ashkenazi and Sephardic Jews from North Africa and Southern Europe in particular, are conducted to ascertain this scenario.

## Supporting information

S1 Table Haplotypes of 51 patients with Parkinson disease carrying the *LRRK2* G2019S mutation. (XLSX)

S2 Table Allele frequencies of 10 microsatellites in 39 Arab-speaking and 38 Berber-speaking control individuals. (XLSX)

S3 Table Mitochondrial DNA polymorphisms for HV1 and HV2 in 29 males carrying the G2019S mutation. A: Arab-speaking; B: Berber-speaking; M: Mixed.
(XLSX)
